# COVID-19 infection analysis framework using novel boosted CNNs and radiological images

**DOI:** 10.1038/s41598-023-49218-7

**Published:** 2023-12-09

**Authors:** Saddam Hussain Khan, Tahani Jaser Alahmadi, Tariq Alsahfi, Abeer Abdullah Alsadhan, Alanoud Al Mazroa, Hend Khalid Alkahtani, Abdullah Albanyan, Hesham A. Sakr

**Affiliations:** 1 Department of Computer Systems Engineering, University of Engineering and Applied Science, Swat, 19060 Pakistan; 2 Department of Information Systems, College of Computer and Information Sciences, Princess Nourah Bint Abdulrahman University, P.O. Box 84428, 11671 Riyadh, Saudi Arabia; 3 Department of Information Systems and Technology, College of Computer Science and Engineering, University of Jeddah, Jeddah, Saudi Arabia; 4 Computer Science Department, Applied College, Imam Abdulrahman Bin Faisal University, Dammam, Saudi Arabia; 5 College of Computer Engineering and Sciences, Prince Sattam Bin Abdulaziz University, Al-Kharj, Saudi Arabia; 6 Nile Higher Institute for Engineering and Technology, Mansoura, Egypt

**Keywords:** Computational biology and bioinformatics, Cardiology, Health care, Medical research, Engineering

## Abstract

COVID-19, a novel pathogen that emerged in late 2019, has the potential to cause pneumonia with unique variants upon infection. Hence, the development of efficient diagnostic systems is crucial in accurately identifying infected patients and effectively mitigating the spread of the disease. However, the system poses several challenges because of the limited availability of labeled data, distortion, and complexity in image representation, as well as variations in contrast and texture. Therefore, a novel two-phase analysis framework has been developed to scrutinize the subtle irregularities associated with COVID-19 contamination. A new Convolutional Neural Network-based STM-BRNet is developed, which integrates the Split-Transform-Merge (STM) block and Feature map enrichment (FME) techniques in the first phase. The STM block captures boundary and regional-specific features essential for detecting COVID-19 infectious CT slices. Additionally, by incorporating the FME and Transfer Learning (TL) concept into the STM blocks, multiple enhanced channels are generated to effectively capture minute variations in illumination and texture specific to COVID-19-infected images. Additionally, residual multipath learning is used to improve the learning capacity of STM-BRNet and progressively increase the feature representation by boosting at a high level through TL. In the second phase of the analysis, the COVID-19 CT scans are processed using the newly developed SA-CB-BRSeg segmentation CNN to accurately delineate infection in the images. The SA-CB-BRSeg method utilizes a unique approach that combines smooth and heterogeneous processes in both the encoder and decoder. These operations are structured to effectively capture COVID-19 patterns, including region-homogenous, texture variation, and border. By incorporating these techniques, the SA-CB-BRSeg method demonstrates its ability to accurately analyze and segment COVID-19 related data. Furthermore, the SA-CB-BRSeg model incorporates the novel concept of CB in the decoder, where additional channels are combined using TL to enhance the learning of low contrast regions. The developed STM-BRNet and SA-CB-BRSeg models achieve impressive results, with an accuracy of 98.01%, recall of 98.12%, F-score of 98.11%, Dice Similarity of 96.396%, and IOU of 98.85%. The proposed framework will alleviate the workload and enhance the radiologist's decision-making capacity in identifying the infected region of COVID-19 and evaluating the severity stages of the disease.

## Introduction

COVID-19, a contagious illness, was first detected in December 2019 and rapidly disseminated across the globe^1,2^. This rapidly spreading infectious disease exhibits a high rate of transmission among humans, leading to faster infection rates and widespread impact. There have been an estimated 675 million suspected cases of COVID-19, resulting in approximately 6.9 million deaths, while around 661 million individuals have recovered from the virus. It is estimated that 99.6% of the infected patients have slight, while 0.4% have severe or critical symptoms^3^. However, it causes respiratory inflammation, difficulty breathing, pneumonia, alveolar damage, and respiratory failure in severe cases, eventually leading to death^4^. Common manifestations of COVID-19 pneumonia include pleural effusion, ground-glass opacities, and consolidation^5^.

COVID-19 detection tests consist of RT-PCR and chest imaging and have been used for clinical evaluation and monitoring^6–8^. Additionally, CT scans are employed in evaluating the severity of COVID-19 and determining appropriate treatment approaches for patients affected by the disease. When a health emergency arises, manually assessing radiological CT scan presents a significant challenge and poses a serious threat for remote regions needing more experienced doctors^9^. Radiological images are usually complex, where the infection region has large size, shape, and position variation. Moreover, the radiological images, obtained through CT imaging, often exhibit complexity and distortion caused by noise inherent in the CT image acquisition process^10,11^.

Automatic detection technology is a serious need to help radiologists improve their performance and deal with many patients and will overcome the burden of manually examining^12,13^. In this regard, Deep Learning (DL)-based diagnostics techniques are implemented in pinpointing COVID-19 CT infection, reducing the radiologist burden for manual assessment and ultimately improving the survival rate^14,15^. The contribution of DL and its capability to classify and segment the image with high accuracy will eliminate the probability of incorrect results by the currently used testing kits^16^. DL will reduce the load on healthcare facilities^17,18^.

The DL-based automated technique's remarkable success in various challenges has gotten researchers' attention for developing medical diagnostic systems^19^. These tools are designed for CT image analysis and facilitate doctors in visualizing lung-related anomalies^20^. These tools have the capability to identify subtle irregularities in COVID-19 patterns that may not be easily discernible through manual examination. However, many studies have utilized pre-existing DL techniques for diagnosing COVID-19, which might not be optimally suitable. These conventional techniques are specifically designed for processing natural images, while the COVID-19 affected regions exhibit distinct radiological patterns that deviate from typical images. Moreover, lung images manifest COVID-19 infection features, such as homogeneity, contrast variation, and structure, which are usually generated by the presence of ground-glass opacities (GGO), water on the lungs (effusion), and consolidation^21^. Therefore, a Convolutional Neural Network (CNN)-based combined detection and analysis scheme is developed that learns COVID-19 patterns to screen and diagnose COVID-19 infectious thoracic radiologic images. The key contributions are as follows:

1. A deep CNN-based analysis system is developed to detect and diagnose lung infections in CT scans and identify the severity of COVID-19. The diagnosis process comprised two phases: detecting COVID-19 infection and conducting an analysis of the lungs using a CT scan.
2. The new Split-Transform-Merge (STM) blocks and Feature Map Enrichment (FME) based deep STM-BRNet detection CNN is proposed to extract and learn various COVID-19 patterns effectively. Additionally, residual multipath learning is integrated into information capacity and boosts the feature-map performance at the final level using transfer learning (TL). These ideas and STM blocks employed multi-path boundary and region-smoothen implementation to capture homogeneous regions, texture variations, and boundary features.
3. A new deep SA-CB-BRSeg segmentation model is proposed to accurately delineate the COVID-19 infectious CT. In this way, average- and max-pooling are employed consistently to leverage COVID-19 infection patterns that pertain to smoothing and discriminative features in encoder-decoder blocks.
4. A novel (Channel Boosted) CB idea is implemented in the proposed SA-CB-BRSeg decoder using TL to learn low contrast and discriminative patterns associated with COVID-19. Moreover, the new attention block is implemented in the SA-CB-BRSeg segmentation CNN to effectively learn mildly infected regions.

The paper follows a structured layout outlined as follows: Section "Related works" offers an in-depth exploration of COVID-19-related studies, providing a comprehensive background. Section 3 introduces the developed COVID-19 infection analysis framework, outlining its key components and functionalities. In Section "Experimental setup", we delve into the material and implementation details, providing specific information on the tools and methodologies employed. Section 5 critically analyzes and discusses the obtained results, highlighting key findings and observations. Finally, in Section "Conclusions", we draw conclusive remarks, summarizing the main insights and contributions of the study.

## Related works

In recent times, CT technology has been widely utilized for diagnosing COVID-19 infection in developed countries like the United States, China, and others. Nonetheless, the manual scrutiny of CT scans poses a substantial and demanding task for radiologists, potentially affecting their overall performance and efficiency. Therefore, an analysis of the region of interest has been performed to detect the position and severity of the infection. Despite the utilization of various conventional techniques for diagnosis, their effectiveness in delivering efficient performance has been limited^22,23^. Therefore, DL-based tools are developed for quick infection analysis and facilitate the radiologist^24,25^. CNN is a sub-type of DL that automatically extracted dynamic features and analyzed COVID-19-infected radiologic images. These models can learn the infected region's valuable features that help detect COVID-19 infection. To address this issue, a range of deep CNNs including VGG-16/19, ResNet-50, Xception, ShuffleNet, and others have been applied to analyze the COVID-19 CT dataset^26^. These models achieved performance from an accuracy of 87% to 98%^20^. Regardless, the previous techniques were utilized for COVID-19 screening but needed more analysis details.

On the other hand, the segmentation of affected regions is widely utilized to precisely identify the location and severity of the disease. Some traditional segmentation approaches were used initially, but they could have delivered better results. Hence, a VB-Net based on CNN was reported to segment infected regions in CT, yielding a dice similarity (DS) of 91%. Additionally, a joint-classification-segmentation (JCS) scheme was utilized to visualize and segment the affected area by combining techniques that reported a DS of (78.3%)^27^. Moreover, the DCN technique exhibited only a marginal level of performance in terms of infected region segmentation, with a DS score of 83.51%^28^. The COVID-19 diagnosis blocks corporate region and edge-based operations and collects diverse features. The technique achieved a 97% detection rate and 93% precision. For the segmentation of lesion infections, a combination of UNet and FPN models was employed, utilizing different encoder-backbone architectures such as DenseNet-ResNet^29^. The detection phase achieved a 99.64% detection rate and 98.72% specificity. Furthermore, an approach incorporating spatial and attention-based U-Net is utilized to enhance feature representation by effectively capturing diverse contextual relationships^30,31^. The majority of previous studies have failed to address these challenges:

- The implementation of reported deep CNN techniques has been conducted on a small dataset. The performance can be improved when evaluating the existing techniques on a large and more diverse dataset.
- Current deep CNN has been utilized for diagnosing COVID-19, which might not be optimally effective for comprehensive COVID-19 analysis.
- It is important to acknowledge that the current diagnosis focuses solely on detecting infected samples, thus lacking information about the different stages of the disease, such as minor, moderate, and severe. Incorporating the ability to classify and differentiate between these stages would provide valuable insights for a more comprehensive understanding of the disease.

## Methodology

**Figure 1:**
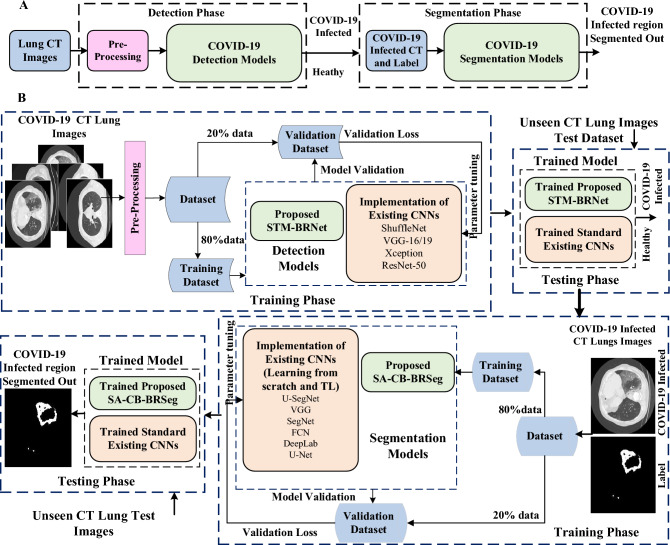
Panel (**A**) outlines the primary stages of the developed two-stage framework, while Panel (**B**) provides a complete workflow in detail.

The deep CNN-based framework is proposed in this study for automated analysis of COVID-19-related abnormalities in the lungs, focusing on infection detection and segmentation. Diagnosing the infectious regions is typically performed through segmentation to explore the infection location and disease severity^32,33^. The diagnostic framework comprises three key technical novelties: (i) the development of the STM-BRNet detection CNN, (ii) the SA-CB-BRSeg segmentation model, and (iii) the utilization of current detection and segmentation CNNs. COVID-19 infected slices are separated from healthy individuals in the detection phase using CT images. While in the segmentation phase, the infectious lesion is segmented to identify the disease severity. Figure 1 illustrates the concise and comprehensive workflows of the developed diagnosis system.

### COVID-19 infection detection

The detection phase of the proposed framework consists of two modules: (i) the STM-BRNet detection CNN model, and (ii) customized existing CNNs for comparative analysis. A novel deep detection CNN has been developed specifically to differentiate COVID-19 infectious lesions from healthy ones. The COVID-19 detection phase is shown in Fig. 1.

#### Proposed detection STM-BRNet

**Figure 2:**
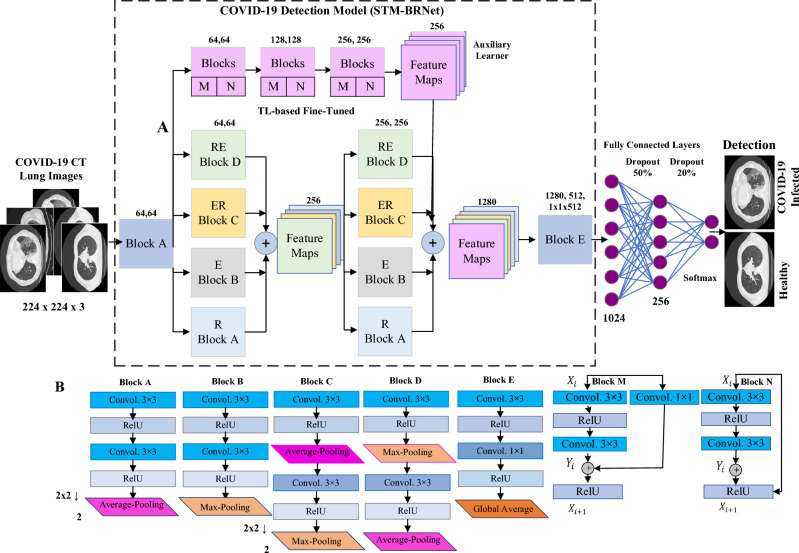
Architectural design for the developed STM-BRNet COVID-19 detection CNN.

This work develops a deep CNN, named “Split Transform and Merge (STM)-BRNet”, that effectively distinguishes COVID-19 infectious CTs from healthy ones, shown in Fig. 2. The proposed STM-BRNet derives its significance from the methodical utilization of innovative STM blocks and Feature map enrichment (FME) ideas. The foundation of STM-BRNet CNN lies in its systematic adoption of dilated convolutions, complemented by region- and edge-based feature processing within STM blocks to capture the smoothing and structure of COVID-19 infected patterns. The STM-BRNet encompasses dilated convolutions that enhance the reception field and preserve data dimensions at the output layer to achieve diverse feature-sets to differentiate infected regions from healthy ones^34^. Moreover, the STM blocks introduce modifications to the novel FME concept, ensuring the preservation of diminished saliency maps, which are subsequently combined to obtain a diverse array of augmented channels and capture minor infection contrast variation. Moreover, the utilization of diverse pooling operations leads to down-sampling, which ultimately strengthens the model's resilience against variations. Additionally, the region operator within the STM block utilizes the average pooling layer for smoothening and noise reduction.

##### Architectural design of the developed STM-BRNet

The STM-BRNet architecture consists of two STM blocks that exhibit an identical topology, strategically arranged to facilitate the learning of different features at both the initial and final levels. The STM is composed of four convolutional blocks, where region and boundary processes are systematically employed. The dimension of each STM boosted block is 256 and 1280, comprised of 26.5 million of the parameters^35,36^. The architecture's primary focus is to capture subtle contrast and texture infection patterns. To achieve this, four distinct blocks, namely Region and Edge (RE), Edge and Region (ER), Edge (E), and Region (R), have been implemented. The dilated convolutional layer, regional/boundary operations, and the Channel Boosted (CB) idea have been modified to effectively learn COVID-19 specific features within each block. The RE block extracts regions and boundaries; it comprises two dilated convolutional layers followed by the average and max-pooling layers, as shown in Eqs. (1–3). Moreover, the ER block extracts edges and regions; it comprises two dilated convolutional layers followed by a max-pooling layer. The E and R blocks learn the edges and smoothness, respectively. Block E generates supplementary feature-maps using TL to attain a variety of channels, while block RE, ER, and E are learning from scratch. The auxiliary channels are created using deep CNNs based on TL. In the merging process within each STM block, these channels are initially squeezed to obtain prominent feature maps.

##### Feature map enrichment (FME)

The complex patterns crucial for distinguishing contrast and texture variations of COVID-19 infected images are learned by the prominent deep CNN based on FME. To systematically enhance the learning process, we employ a sophisticated stacking approach that integrates TL-based residual learning with M and N blocks. Residual CNN designs possess distinct capabilities for feature learning and produce numerous channels that capture information across multiple levels. By strategically concatenating these blocks at the final stage, we are able to effectively explore and learn diverse feature spaces. The integration of these diverse abstractions, acquired from multiple channels, can significantly improve both global and local representations of an image. The original channel blocks are combined with auxiliary channels, the result is a novel concept—an intelligent feature-based ensemble. This innovative arrangement is built upon three sequential residual blocks, enabling us to acquire a wide range of essential features. To further facilitate this robust learning process, we progressively increase the number of channels from 64 to 256. In this ensemble, a single learner makes the ultimate decision, informed by an analysis of diverse image-specific patterns. This deliberate augmentation ensures a comprehensive and refined learning experience, leading to improved quality of outcomes.

These processes enhance the boundary information and region-specific properties, whereas dilated convolutional operations aid in learning the global receptive features. The implementation of multipath-based STM blocks allows for the perception of diverse features, enabling the dynamic capture of minor representative and textural variation information from the COVID-19 infected CT. Additionally, the inclusion of fully-connected and dropout layers helps store crucial features and mitigates the risk of overfitting.

1 $${{\text{x}}}_{{\text{k}},{\text{l}}}= \sum_{{\text{i}}=1}^{{\text{m}}}\sum_{{\text{j}}=1}^{{\text{n}}}{{\text{x}}}_{{\text{k}}+{\text{i}}-1,{\text{l}}+{\text{n}}-1}{{\text{f}}}_{{\text{i}},{\text{j}}}$$

2 $${{x}^{max}}_{k,l}= {max}_{i=1,\dots ,w,j=1,\dots ,w}{x}_{k+i-1,l+j-1}$$

3 $${{x}^{avg}}_{k,l}=\frac{1}{{w}^{2} } \sum_{i=1}^{w}\sum_{j=1}^{w}{x}_{k+i-1,l+j-1}$$

4 $${{\text{x}}}_{{\text{Boosted}}=}{\text{b}}\left( {{\text{x}}}_{{\text{ER}}}|| {{\text{x}}}_{{\text{RE}}}{||{\text{x}}}_{{\text{R}}}{||{\text{x}}}_{{\text{E}}}\right)$$

5 $${\text{x}}={\sum }_{{\text{a}}}^{{\text{A}}}{\sum }_{{\text{b}}}^{{\text{B}}}{{\text{v}}}_{{\text{a}}}{{\text{x}}}_{{\text{Boosted}}}$$

6 $$\upsigma \left({\text{x}}\right)=\frac{{{\text{e}}}^{{{\text{x}}}_{{\text{i}}}}}{\sum_{{\text{i}}=1}^{{\text{c}}}{{\text{e}}}^{{{\text{x}}}_{{\text{c}}}}}$$

The feature-map and dimension are represented by 'x' and 'k x l', respectively. While Eq. (1) depicts the kernels and size represented by 'f' and 'i x j'. In contrast, the output ranges to [1 to k-m + 1, l-n + 1]. Moreover, the pooling operation window size is represented by **w,** respectively, on convolved output $({{\text{x}}}_{k,l}$) (Eqs. 2–3). In Eq. (4), the feature-maps of block RE, ER, and R are signified by ${{\text{x}}}_{{\text{RE}}}$, ${{\text{x}}}_{{\text{ER}}}$, and ${{\text{x}}}_{{\text{R}}}$, respectively. Likewise, the auxiliary feature-maps of block R achieved using TL are denoted as ${{\text{x}}}_{{\text{E}}}$. These channels are boosted by concatenation operation b(.). The neuron quantity and activation in Eq. (6) are shown with ${{\text{v}}}_{{\text{a}}}$. and $\upsigma$.

#### Implementation of existing detection CNNs

In recent times, CNNs have shown remarkable effectiveness in detecting and segmenting medical images within the field of medicine^19^. The detection phase utilizes various models including VGG-16/19, ResNet-50, ShuffleNet, and Xception, among others^37^. These deep CNNs with varying in-depth and network designs are tailored to screen and analyze infectious regions.

### COVID-19 infected regions segmentation

The proposed STM-BRNet aims to classify COVID-infected patients from healthy patients by utilizing the capabilities of deep CNN architectural ideas. The infected images are provided the segmentation CNNs for delineating COVID-19 infection regions that identify the disease's severity. This paper implements two different experimental setups for infection segmentation: (i) proposed SA-CB-RESeg segmentation, (ii) target-specific segmentation CNNs implementation from scratch, and Transfer Learning (TL).

#### Proposed SA-CB-RESeg segmentation CNN

We propose a new fine-grained pixel-wise segmentation approach known as SA-CB-RESeg. The SA-CB-RESeg CNN architecture consists of two encoders and boosted decoder blocks, specifically designed to enhance the learning capacity of SA-CB-RESeg. To achieve this, a systematic combination of average-pooling, max-pooling, and convolutional operations is employed in both the encoding and decoding stages. This enables the network to efficiently learn the properties associated with regions and boundaries of COVID-19 infected areas^14,38^. Furthermore, through the convolutional operation, trained filters are applied to the images, resulting in the generation of feature maps that effectively capture unique and discernible patterns. The encoders and decoders are symmetrically designed, with a total of 21.2 million learning parameters. In the encoder, max-pooling is utilized for down-sampling purposes during pooling operations. Conversely, in the decoder, an un-pooling operation is employed to perform up-sampling. Lastly, the convolutional layer is utilized to categorize COVID-19 and background pixels.

**Figure 3:**
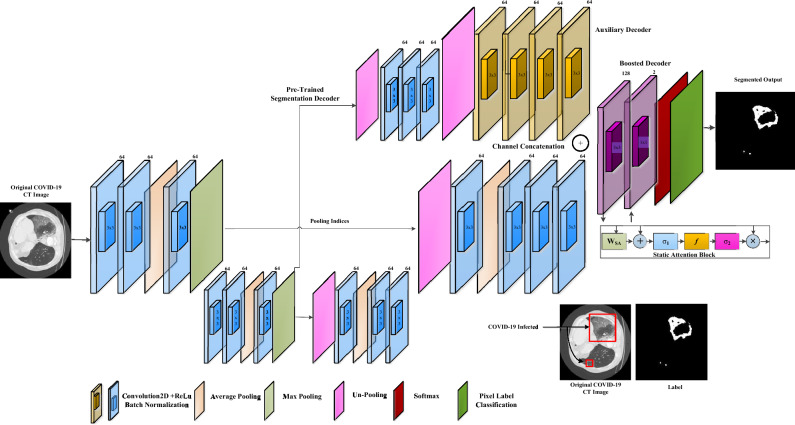
Architectural design for the proposed SA-CB-RESeg.

The encoder is designed to learn semantically meaningful COVID-19 specific patterns. However, the encoder loses spatial information essential for infected region segmentation because it reconstructs the infection map. In this regard, to retain the spatial information from the corresponding encoders, decoders are utilized by leveraging pooling indices. These positional indices are stored in each pooling operation and are helpful for reconstruction and mapping on the decoder side. Moreover, the pooling operation performs down-sampling and reduces the spatial dimension (Fig. 3).

##### Boosting significance

The new CB idea is introduced by concatenation the original feature maps of the decoder with additional feature maps through TL to improve learning the minor contrast COVID-19 infected region. The developed SA-CB-RESeg utilized the additional channels generated from pre-trained CNN using TL combined with the original to get rich information feature maps and improve generalization. The SA-CB-RESeg benefited from learning from scratch and tuning on COVID-19 images using TL and CB. The boosting channels increase the SA-CB-BRSeg representative’s capacity. Moreover, ${\mathbf{X}}_{{\text{RE}}-{\text{e}}}$ and ${\mathbf{X}}_{{\text{RE}}-{\text{d}}}$ refer to the encoder (e) and decoder (d) blocks utilized in the SA-CB-RESeg model, as depicted in Eqs. (7) and (8). Consequently, Eq. (9) illustrates the process of boosting and auxiliary channel (AC) process performed at the decoder side.

7 $${{{\varvec{X}}}_{RE-e}=f}_{c} ({{\varvec{x}}}^{avg} ||{{\varvec{x}}}^{max} )$$

8 $${{\mathbf{X}}_{{\text{RE}}-{\text{d}}}=f}_{c}\left({\mathbf{x}}^{{\text{max}}} ||{\mathbf{x}}^{{\text{avg}}}\right)$$

9 $${\mathbf{X}}_{\mathrm{CB }}=b\left( {\mathbf{X}}_{{\text{RE}}-{\text{d}}}|| {\mathbf{X}}_{{\text{AC}}}\right)$$

##### Static attention

**Figure 4:**
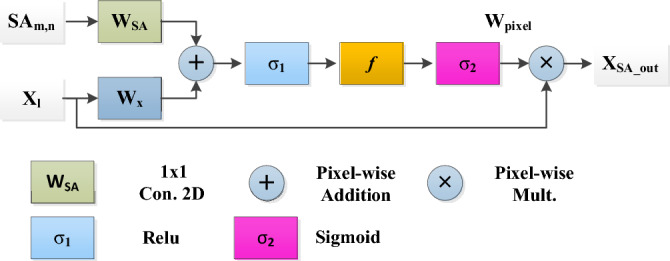
Static attention block designing.

Static attention (SA) enhances the learning capability of the COVID-19-infected areas by locating high weightage^39^. The SA block detail is shown in Fig. 4. ${X}_{l}$ indicates the input map and ${W}_{pixel}$ is the weighted-pixel having a range of [0, 1] (Eq. (10)). The result ${X}_{SA\_out}$ emphasizes the affected region while minimizing the presence of unrelated characteristics. In Eqs. (11) and (12),${\sigma }_{1}$ and ${\sigma }_{2}$ is the activation, ${b}_{SA}$ and ${b}_{f}$ is biasness, and ${W}_{x}$, ${W}_{SA}$, $f$ is the transform, respectively.

10 $${\mathbf{X}}_{SA\_out}={W}_{pixel}.{\mathbf{X}}_{l}$$

11 $${X}_{relu}={\sigma }_{1}\left({W}_{x}{\mathbf{X}}_{l}+{W}_{SA}{SA}_{m,n}+{b}_{SA}\right)$$

12 $${W}_{pixel}={\sigma }_{2}(f({X}_{relu})+{b}_{f})$$

#### Existing segmentation CNNs

To effectively segment the COVID-19 infected region in CT scans, several deep CNNs have been employed, utilizing diverse datasets^40^. This study employs current DeepLab, U-SegNet, SegNet, VGG, U-Net, nnSegment Anything Model (SAM)^41^, nnUNet^41^, and FCN segmentation CNNs^42–44^. The nnSAM model utilizes the robust feature extraction capabilities inherent in SAM, harnessing its strength and effectiveness. The existing segmentation CNNs have been implemented for comparative studies. In our study, we have utilized existing CNN models through two approaches: training from scratch and weight initialization. To benefit from the knowledge gained by pre-trained CNNs, we employ TL by initializing the weights from these models^45^. This allows us to leverage the learned features and patterns from the pre-training stage. Subsequently, we fine-tune these weights using CT images specific to our study. This combination of TL and fine-tuning enables our models to effectively capture the relevant features and optimize their performance for CT image analysis.

## Experimental setup

### Dataset

**Figure 5:**
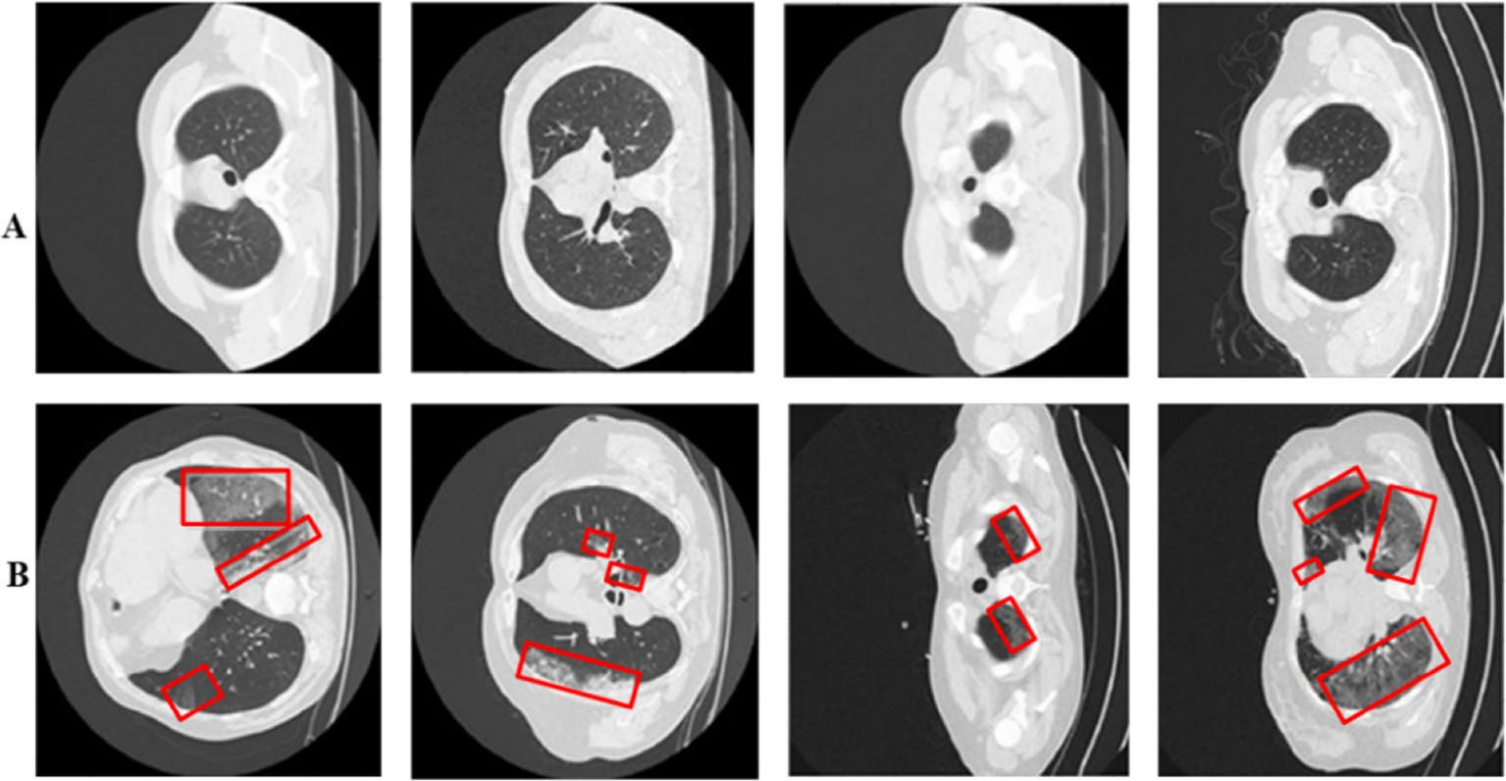
Panels (**A**,**B**) represent COVID-19 infected vs. Healthy samples. While the infected regions are red highlighted.

Chest CT scans are highly effective in diagnosing COVID-19 due to their high sensitivity. One of the key advantages of using CT scans is their ability to enhance the visibility of internal anatomy by eliminating overlapping structures. This characteristic enables a more accurate analysis of the impacted regions within the lungs, contributing to a more precise examination. In our research, we make use of a dataset provided by the SIRM^46^. This dataset comprises CT lung images from 30 patients, encompassing a total of 2684 images. The dataset includes both COVID-19 infected (Fig. 5B) and healthy (Fig. 5A) patients, with corresponding labels available in .nii.gz format, as shown in Fig. 5. To ensure the accuracy of the dataset, an experienced radiologist carefully examined each image. Furthermore, the radiologist provided binary labels indicating the presence of infected lung regions, allowing for the identification and analysis of these areas.

### Implementation details

The detection and segmentation CNNs are trained independently within our developed diagnostic system. We have meticulously assembled a dataset encompassing CT lung images from both COVID-19 infected patients and individuals in healthy conditions. We have categorized COVID-19 infected and healthy classes to effectively utilize the dataset. The COVID-19 infected class comprises 1362 images, while the healthy class contains 1322 images. However, for segmentation, we specifically employ the class of images representing COVID-19 infected cases (1322 images) along with their corresponding labels., as they provide valuable insights for analyzing the infected area. To ensure effective training and evaluation, we divided them into distinct training and testing sets. To maintain consistency, we employed an 80:20% ratio for the diagnosis phases. Furthermore, to enhance the training process, we employ cross-validation techniques to divide the training set into separate training and validation subsets. This approach enables robust evaluation and validation of our model's performance. Our proposed novel architecture, which utilizes Deep CNNs for detection and segmentation, is implemented using MATLAB 2022a. The simulations are performed on an NVIDIA GTX-T computer equipped with 64 GB of memory, enabling efficient and accurate performance throughout the process. Each model takes almost 13–23 h. ~ 1.5–2.5 h. /epoch, during training. Hyper-parameters control the deep CNNs' optimization and convergence. To ensure smoothing and efficient convergence, CNN models undergo training for 10 epochs with carefully selected optimal hyper-parameters^47^. The hyperparameters used in the experiment: a learning rate of 10^−3^, epochs (10), model optimizer set to SGD, batch size (16), linear momentum (0.90), and cross-entropy as a loss function. The cross-entropy loss function is commonly used for probability SoftMax-based classification and pixel-wise segmentation to measure the dissimilarity between predicted and ground truth labels.

### Performance evaluation

The performance of the developed framework is evaluated using standard measures, which are presented in Table 1. The detection metrics, including accuracy, recall, etc., are depicted in Eqs. (13–17). In the evaluation of segmentation CNNs, the IoU (Intersection over Union) and DS (Dice Similarity) coefficients are utilized, as represented in Eqs. (18) and (19), respectively. The segmentation accuracy (S_Acc) refers to the precise prediction of pixels corresponding to infected and healthy regions. The DS coefficient is employed to measure structural similarity, while the IoU is utilized to assess the overlapping ratio between the predicted and original images.

**Table 1:** Detail of performance measures.

Measure	Symbol	Detail
Accuracy	Acc	The ratio of accurate detections to the total number of predictions
Recall	R	Correct prediction ratio (Positive)
Specificity	S	Correct predictions ratio (Negative)
Precision	P	Correct predictions ratio (Positive) in overall predictions
Mathew Correlation Coefficient	MCC	Evaluate the efficacy of the confusion matrix for imbalanced data
Jaccard Coefficient	IoU	% Degree of similarity between labeled and detected regions
Dice-Similarity	DS	% Degree of weighted similarity between labeled and detected areas
Segmentation-Acc	S_Acc	% Accurate pixel partitioned into COVID-19 and Background

13 $$Acc=\frac{Correctly Predicted Slices}{Total Slices}\times 100$$

14 $$p=\frac{Correctly Predicted COVID-19 }{Correctly Predicted COVID-19+Incorrectly Predicted COVID-19}$$

15 $${\text{R}}=\frac{\mathrm{Accurately Detected COVID}-19}{\mathrm{Total COVID}-19}$$

16 $$S=\frac{Correctly Predicted Healthy}{Total Healthy }$$

17 $$F-Score=2\frac{\left(P x R\right)}{P+R}$$

18 $$IoU=\frac{predicted infected region \cap Labeled infected region}{predicted infected region \cup Labeled infected region}$$

19 $$DS Score=\frac{2*Correctly Predicted infected region}{2*Correctly Predicted infected region+Total infected region}$$

## Results

The present study introduces a novel two-stage diagnostic framework for examining infectious regions in the lungs associated with COVID-19. Distributing the proposed into two stages has two main advantages: improving the performance and reducing the computational complexities. Moreover, screening of COVID-19 infected samples and then analyzing the infectious region helps in the diagnosis of the disease. Moreover, the two-phase approach bears similarity to the conventional clinical procedure of recommending further diagnostic examinations for suspects after the initial detection. The performance of the proposed STM-BRNet and SA-CB-RESeg CNNs are evaluated based on standard performance metrics. The proposed models are evaluated on test data and indicate considerable performance.

### Detection stage analysis

**Figure 6:**
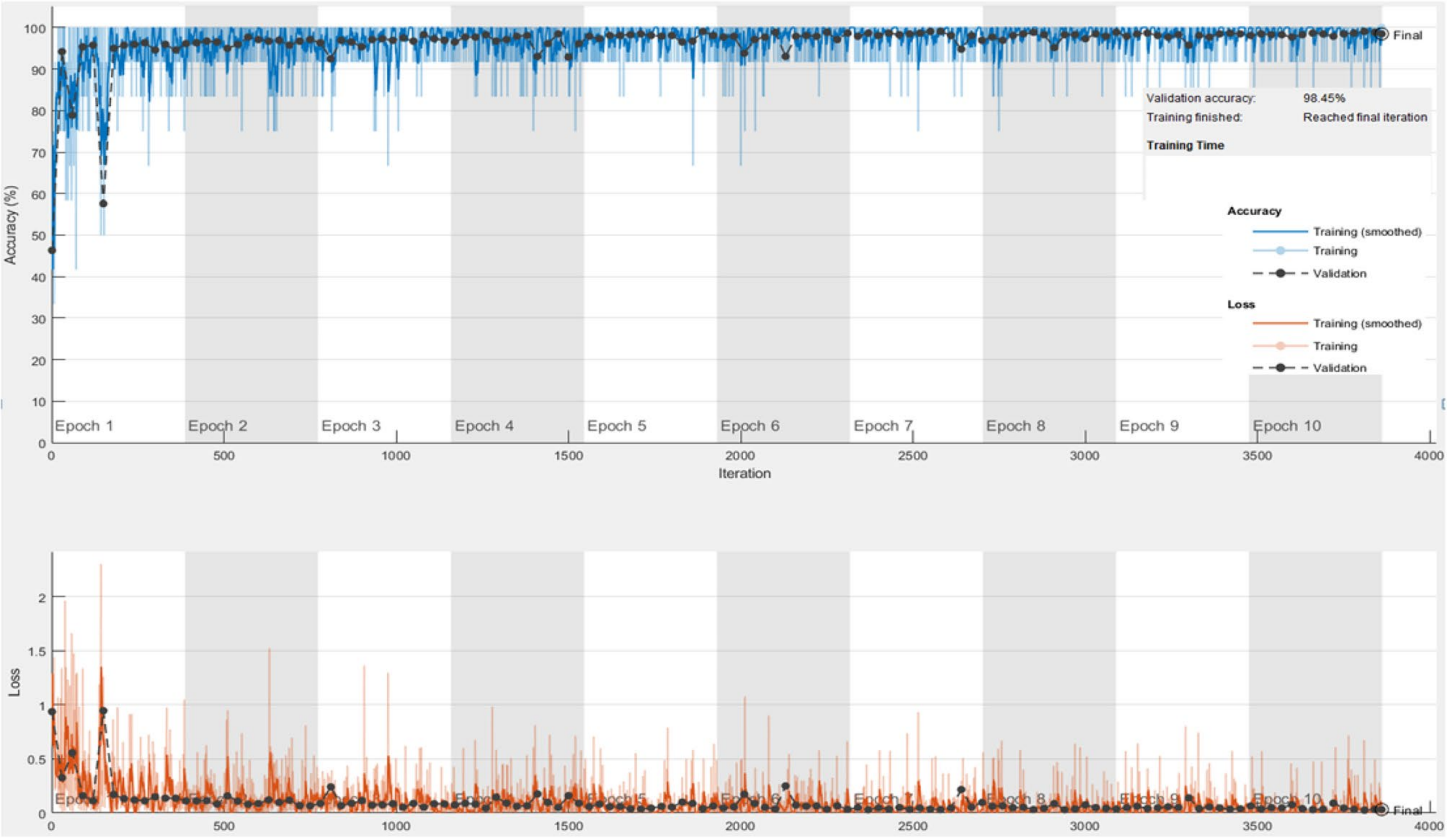
Training convergence plot of the developed STM-BRNet detection CNN.

We have evaluated the screening capability of the proposed STM-BRNet in identifying COVID-19 specific CT scans and compared it with existing CNNs in this stage. The detection stage is improved to achieve a high sensitivity rate in identifying the characteristic pattern of COVID-19 while minimizing the occurrence with fewer false positives (shown in Table 2). The learning plot indicates accuracy and loss for the validation dataset of the developed STM-BRNet CNN (Fig. 6). High training and validation error at the start has a maximum error; SGD fluctuates heavily. At the end of the training, SGD movement becomes smooth and reaches the solution.

**Table 2:** Performance analysis of the developed STM-BRNet and current models.

CNNs	Acc	F-score	ROC_AUC	PR_AUC	Pre	MCC	Spec	Rec
ShuffleNet	92.26	92.44	96.4	96.36	91.38	81.87	90.96	91.18
VGG-19	94.35	94.43	98.33	98.84	94.15	87.21	93.98	92.94
Xception	95.83	95.81	99.09	98.58	97.56	90.67	96.99	91.67
VGG-16	96.13	96.12	98.45	98.93	97.58	91.52	97.59	92.35
ResNet-50	96.73	96.77	99.26	98.76	96.49	92.02	96.39	94.71
Proposed STM-BRNet	98.01	98.11	99.67	99.10	98.09	94.85	98.01	98.12
Ablation study of the Proposed combination (Cbn.) of Blocks								
E Blocks (Cbn.)	94.84	94.74	98.65	98.93	97.04	89.64	97.19	92.55
R Blocks (Cbn.)	95.19	96.23	99.56	98.96	95.46	90.19	95.48	94.91
E & R/B &R Blocks (Cbn.)	96.23	96.22	98.55	98.93	97.78	91.72	97.79	94.91
E + R + ER/BR (Cbn.)	97.12	97.12	99.27	98.90	97.28	94.25	97.39	96.86
Comparison with the reported Detection Techniques								
JCS^27^	–	–	–	–	–	–	93.00	95.00
VB-Net^48^	–	–	–	–	–	–	90.00	87.00
DCN^28^	–	96.74	–	–	–	–	–	–
3DAHNet^49^							90.00	85.00

#### STM-BRNet’s performance analysis

The performance of the developed STM-BRNet is evaluated on the test set using various performance measures, including Accuracy, F-score, MCC, etc. Comparing STM-BRNet with the existing ResNet-50 model, it demonstrates favorable generalization in terms of accuracy (STM-BRNet: 98.01%, ResNet-50: 96.73%), and F-score (STM-BRNet: 98.11%, ResNet-50: 96.77%). The STM-BRNet technique, which incorporates edge and region-based STM blocks and FME using TL, significantly improves the detection rate by correctly classifying a higher number of samples as true positives. The high-intensity channel plays a crucial role in highlighting boundaries, while the approximation maps provide a more intricate representation of the region. The fusion technique employed in this study emulates the concept of sharpening an image using Laplacian of Gaussian, enabling the preservation of optimal characteristics of infectious regions. Additionally, the CB idea facilitates the learning of diverse feature maps from the pre-trained scenario, effectively capturing texture variations. The systematic implementation of these ideas yields enhanced performance, as evidenced by improvements in metrics such as accuracy, MCC, and F-score (as presented in Table 2). Moreover, TL-based residual learning systematically extracts features, starting with basic image-level features such as edges and advancing to more intricate texture-based differences. Moreover, the ablation studies have been added to provide valuable insights into the individual contributions of systematic arrangement blocks to the overall performance of the proposed STM-BRNet model. These ablation experiments will provide a more comprehensive understanding of the role of each concept of blocks in the STM-BRNet and help us identify the several variations of blocks that are most influential in improving detection performance. This analysis enhances the robustness and contributes to a deeper understanding of the STM-BRNet architecture's effectiveness. Furthermore, the performance of the STM-BRNet is further increased by adding fully-connected layers to emphasize the learning and improve the generalization.

#### Performance analysis with the existing CNNs

We conducted a comparative evaluation to assess the performance of the developed STM-BRNet against five customized detection CNNs, namely VGG, ResNet-50, Xception, and ShuffleNet. These CNNs are well-known for their ability to tackle complex problems and have proven effective in detecting anomalies in lung images. To ensure a fair comparison, the customized CNNs were trained on COVID-19 specific images. In contrast, the proposed STM-BRNet demonstrated superior performance and achieved higher scores in F-score, MCC, accuracy, and other metrics when compared to the customized CNNs on the test dataset. This can be observed in Table 3 and Fig. 9, where the STM-BRNet outperforms the other models.

**Table 3:** Performance analysis of the developed and existing segmentation CNNs (trained from scratch).

Model	Region	DSC	Acc	IoU	BF	Global-Acc	Mean-Acc	Mean-IoU	Weighted-IoU	Mean-BF
Ablation study of the proposed segmentation CNNs										
Proposed SA-CB-BRSeg	Infected	96.40	99.21	98.85	99.09	99.51	99.49	98.98	99.09	98.32
	Background	99.02	99.72	99.31	97.45					
Proposed CB-BRSeg	Infected	95.96	99.01	98.43	98.87	99.25	99.17	98.69	98.80	98.03
	Background	98.90	99.48	99.09	97.33					
Proposed SA-BRSeg	Infected	95.61	98.83	98.35	98.47	98.99	98.97	98.46	98.57	97.42
	Background	98.40	99.38	98.85	96.73					
Comparative studies with the existing CNNs										
Deeplabv3	Infected	95.00	98.48	97.59	97.53	99.03	98.91	98.14	98.33	97.08
	Background	98.30	99.33	98.67	96.39					
nnSAM^41^	Infected	94.85	98.33	97.45	97.39	98.93	98.81	98.04	98.23	96.88
	Background	98.27	99.30	98.64	96.36					
U-SegNet	Infected	94.65	98.25	97.01	97.02	98.82	98.73	97.52	97.68	96.52
	Background	98.01	99.16	98.10	95.22					
nnUNet	Infected	94.63	98.20	96.09	96.98	98.80	98.69	97.49	97.65	96.49
	Background	98.27	99.18	98.12	95.27					
SegNet	Infected	94.30	98.97	96.56	96.73	98.72	98.71	97.18	97.32	96.48
	Background	97.90	98.45	97.79	95.22					
U-Net	Infected	94.00	98.61	95.98	96.91	98.40	98.44	96.70	96.87	95.49
	Background	97.70	98.28	97.42	94.07					
VGG-16	Infected	91.00	91.38	88.91	89.37	95.59	94.81	91.05	91.53	83.66
	Background	95.00	98.25	93.18	77.95					
FCN-8	Infected	90.70	90.92	89.11	87.74	95.32	94.55	90.63	90.20	82.43
	Background	94.00	98.18	92.15	77.11					
Comparative studies with reported segmentation techniques										
VB-Net^48^	Infected	91.00	–	–	––	–	–	–	–	–
Weakly Sup ^50^	Infected	90.00	–	––	–	–	–	–	–	–
MTL^51^	Infected	88.00	–	–	–	–	–	–	–	–
DCN^28^	Infected	83.50	–	–	–	–	–	–	–	
U-Net-CA^30^	Infected	83.10	–	–	–	–	–	–	–	–
Inf-Net^52^	Infected	68.20	–	–	–	–	–	–	–	–

Gl-Acc, Mn-Acc. represents global and mean accuracy where Mn-IoU and Wt-IoU denote means and weighted IoU.

#### Features visualization and PR/ROC analysis

**Figure 7:**
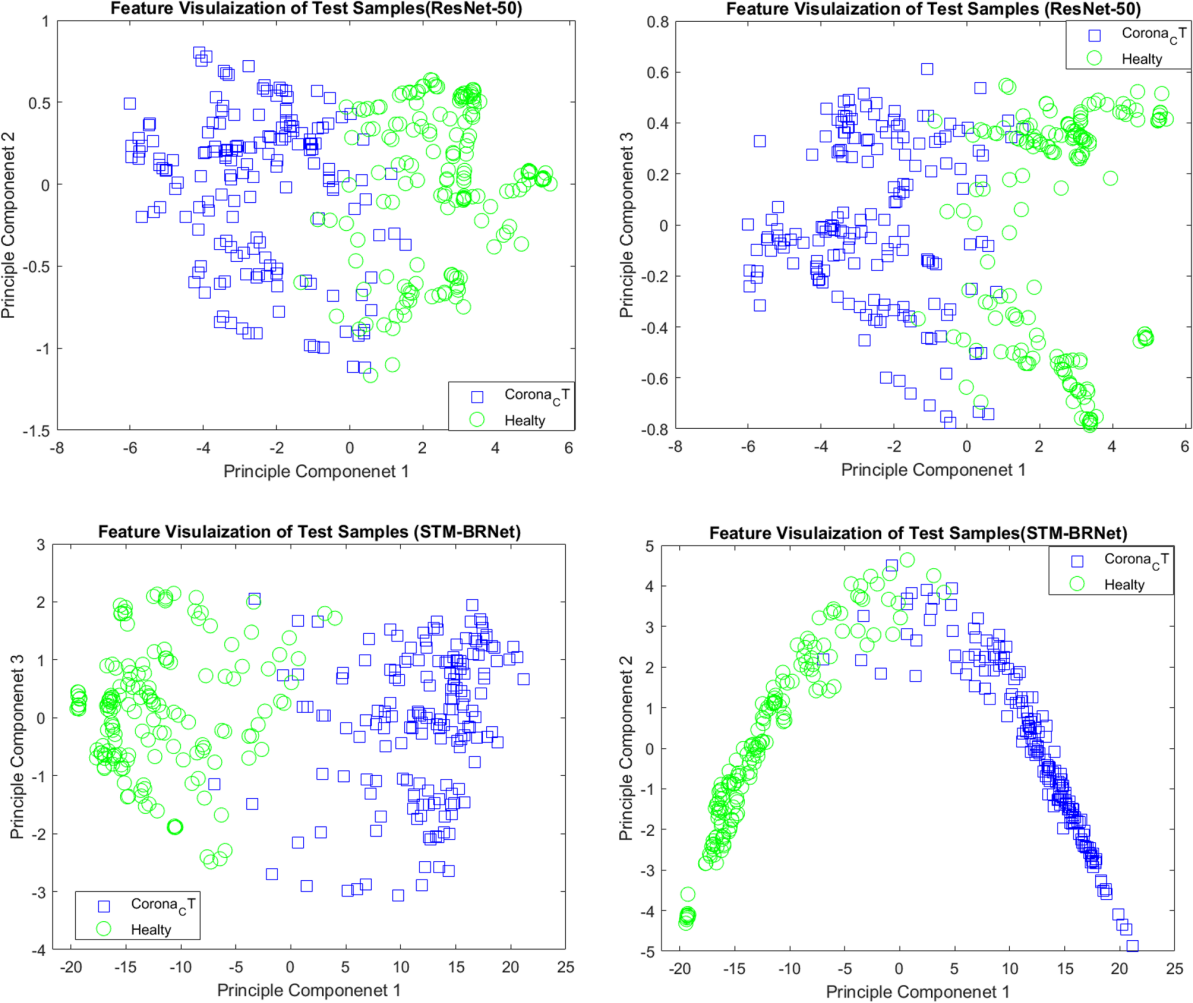
Feature Visualization of the developed STM-BRNet and ResNet-50 for the first, second, and third principal components generated.

**Figure 8:**
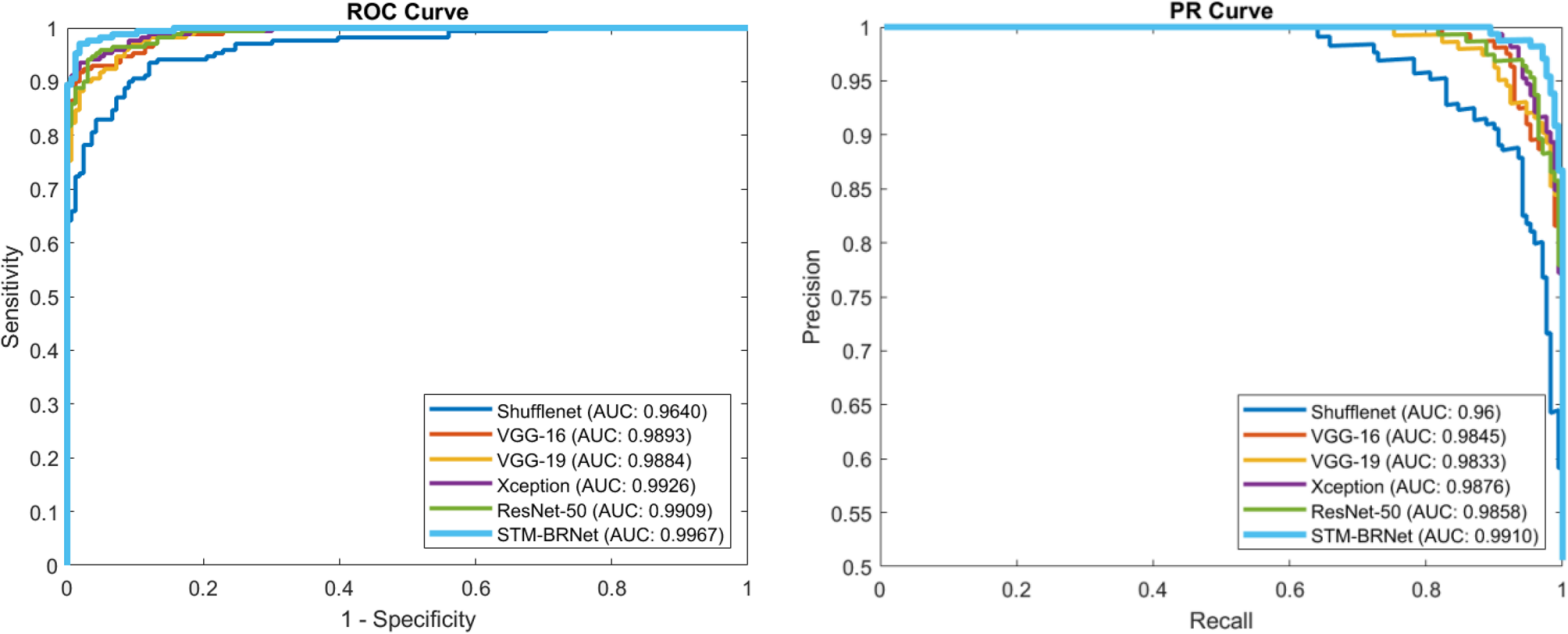
PR and ROC curve for the developed STM-BRNet and current CNNs.

The significant detection capability of STM-BRNet is effectively demonstrated through the principal component analysis (PCA) plot. PCA allows for the reduction of dimensionality in STM-BRNet features, enabling the identification of distinct patterns for enhanced discrimination. Figure 7 provides a comparison of deep feature-based analysis between STM-BRNet and the best performing existing ResNet-50 model. The PCA plot, incorporating the first, second, and third principal components, clearly showcases the remarkable learning ability of STM-BRNet. Additionally, a quantitative assessment of the discrimination ability is conducted through the use of detection rate curves (PR/ROC), as illustrated in Fig. 8. Through a comprehensive evaluation of various threshold configurations, these performance measurement curves provide valuable insights into the generalization capabilities of STM-BRNet by examining COVID-19 infected and healthy individuals. Furthermore, STM-BRNet exhibits impressive learning ability when compared to different CNNs, particularly at the optimal threshold. The PR and ROC curves, generated based on STM-BRNet features, yield a significantly higher area AUC, indicating superior model performance in COVID-19 infection screening. This higher AUC value reflects the heightened accuracy and effectiveness of the STM-BRNet model.

### Infected region analysis

CTs infected are separated using the developed STM-BRNet and assigned to segmentation CNN to analyze the infection severity. The infected slices and normal have minor contrast variations in the early stage. However, isolating the infected region from the healthy region is quite challenging. Hence, the proposed SA-CB-RESeg model effectively segments infection boundaries with minimal contrast variation. Additionally, conducting a region analysis becomes crucial in assessing the disease severity, categorizing it as mild, moderate, or severe, and designing appropriate treatment strategies.

#### The developed SA-CB-RESeg segmentation evaluation

The developed SA-CB-BRSeg analyzes COVID-19 infectious areas in CT images. The existing segmentation CNNs have been optimized based on COVID-19 infected specific patterns and imagery features. The experimental results on unseen data show the significance of the proposed SA-CB-BRSeg (Table 3). Moreover, Furthermore, COVID-19 infection patterns exhibit variability across different patients. Our proposed SA-CB-BRSeg model excels in performing precise pixel-wise segmentation of infected regions, resulting in the generation of high-quality maps. In subjective evaluations (refer to Figs. 10 and 11), our model surpasses existing CNNs.

The SA-CB-BRSeg introduces an innovative approach that combines region-homogeneity and boundary-based implementation, utilizing average and max-pooling techniques. This systematic implementation, in conjunction with TL and CB methods, enables the model to accurately capture well-defined boundaries and texture variations within the infected lung region. Additionally, a comprehensive analysis of the infected region provides valuable insights into the patterns of infection and their impact on surrounding organs. The obtained results showcase the exceptional learning capability of the proposed SA-CB-BRSeg in accurately capturing COVID-19 infection patterns. This is evidenced by impressive metrics such as a DS score and IoU of 96.40% and 98.85% respectively (refer to Table 3). Furthermore, the model demonstrates its ability to precisely learn discriminative boundaries, achieving a higher BFsa value of 99.09%.

#### Segmentation analysis with the existing CNNs

**Figure 9:**
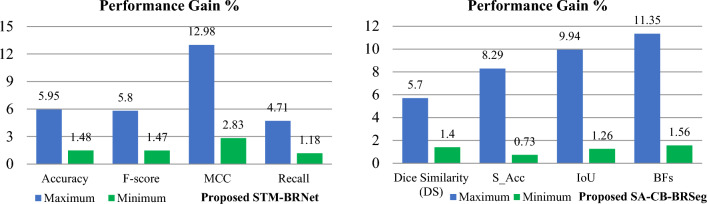
The developed STM-BRNet and SA-CB-BRSeg performance gain over the current CNNs.

**Figure 10:**
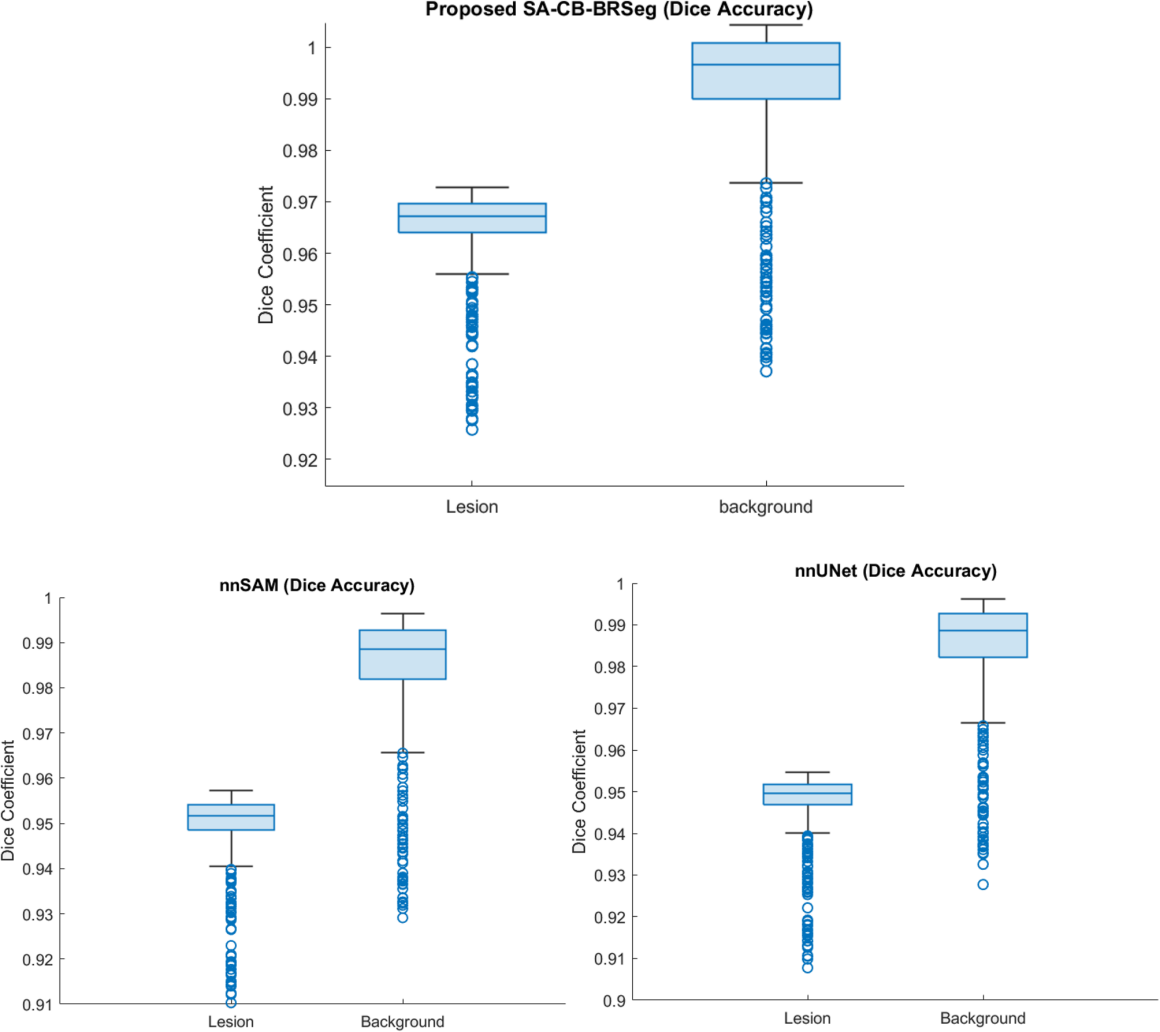
Dice Score of the developed SA-CB-BRSeg and existing nnSAM and nnUNet segmentation CNNs results.

**Figure 11:**
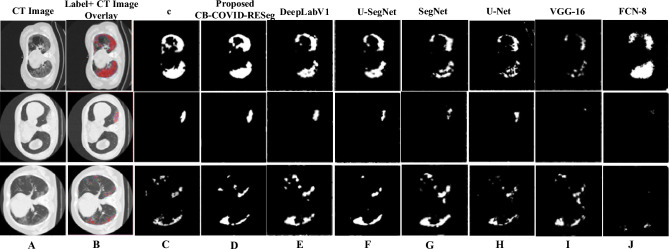
Visual analysis of the developed SA-CB-BRSeg and existing segmentation CNNs results.

The existing segmentation CNNs are employed to assess the learning capacity of the developed SA-CB-BRSeg. Moreover, the performance of SA-CB-BRSeg is compared with six widely recognized segmentation CNNs for a comprehensive evaluation (DeepLabv3, nnSAM^41^, U-SegNet, nnUNet^41^, SegNet, U-Net, VGG-16, and FCN) (Table 3 and Figs. 9, 10). Figure 11 illustrates the segmented infectious regions achieved by the proposed SA-CB-BRSeg as well as existing CNN models. The quantitative analysis recommends that the developed SA-CB-BRSeg performs better than existing segmentation CNNs. However, the results show that customized CNNs perform poorly in learning mildly infectious regions. In contrast, VGG-16, FCN, and U-Net CNNs fluctuate in various stages of CT images and show less robustness in the models. The maximum accuracy in existing segmentation CNNs (Deeplabv3) is (98.48%) for the infected region. Consequently, the DS and IOU are (95% against 96.40%) and (97.59% beside 98.85%), respectively.

The SA-CB-BRSeg method demonstrates its potential suitability for regions with varying degrees of infection, ranging from moderate to severe. Moreover, the proposed and existing models' performance is improved using radiological and augmented data. The SA-CB-RESeg model, which we have developed, exhibits low complexity yet achieves greater accuracy compared to highly complex models with larger depths. Through the incorporation of pixel-wise distribution, the SA-CB-RESeg model significantly improves the accuracy of segmentation across various stages of infected regions.

#### TL based evaluation

**Figure 12:**
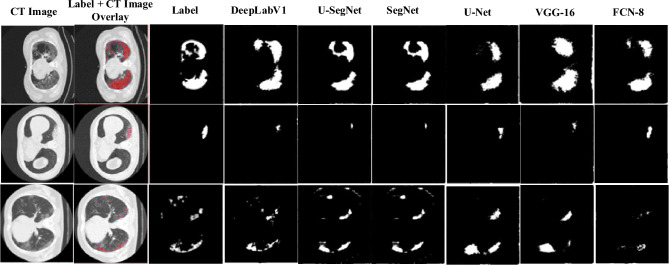
Visual evaluation of TL-based segmentation CNNs results on the test set.

The performance of TL-based fine-tuning demonstrates an improvement over training the model from scratch, as evident in the significant gains observed in various metrics. The DS score shows an increase from 0.35% to 4%, S-Acc improves from 0.28% to 7.37%, the DS score rises from 0.44% to 8.13%, and BFs increase from 0.66% to 9.34%, as illustrated in Tables 3 and 4. These performance gains strongly suggest that TL enhances the overall performance of the model compared to learning from scratch. To achieve this, TL-based feature maps are generated and seamlessly integrated into the decoder of the developed SA-CB-RESeg model (Fig. 12). By leveraging TL, the model gains advantages from fine-tuned weights and acquired patterns from pre-existing trained scenarios, leading to improved convergence and generalization capabilities^53,54^. Moreover, the radiologist labeled and augmented data are combined to improve the developed SA-CB-RESeg performance^55^. The analysis of infected regions is achieved using the best-performing existing TL-based trained DeepLabv3 CNN for comparative analysis. The DeepLabv3 has gained accuracy (98.76%) and IOU (98.03%) for infectious regions (Table 4).

**Table 4:** Performance of segmentation CNNs (implemented using TL).

Model	Region	DSC	Acc	IoU	BF	Global-Acc	Mean-Acc	Mean-IoU	Weighted-IoU	Mean-BF
Deeplabv3	Infected	95.35	98.76	98.03	97.71	99.23	99.15	98.40	98.48	97.19
	Background	98.65	99.53	98.76	96.66					
U-SegNet	Infected	95.20	98.41	97.52	97.46	98.93	98.81	98.10	98.25	96.94
	Background	98.60	99.67	98.61	96.32					
SegNet	Infected	95.10	98.29	97.70	97.41	99.10	98.95	98.09	98.22	96.82
	Background	98.10	99.62	98.55	96.23					
U-Net	Infected	94.90	98.74	97.62	98.19	99.22	99.06	98.06	98.15	96.63
	Background	98.20	99.07	98.49	95.86					
VGG-16	Infected	94.80	98.29	97.07	97.11	98.89	98.79	97.61	97.74	96.18
	Background	97.90	99.23	98.24	95.29					
FCN-8	Infected	94.70	98.29	97.04	97.08	98.87	98.76	97.59	97.71	96.16
	Background	98.00	99.21	98.15	95.27

#### Visual analysis of the proposed SA-CB-BRSeg

Visual The deep SA-CB-BRSeg model is utilized for visual analysis of COVID-19 infection segmentation, enabling the identification and examination of infected regions. The subjective evaluation demonstrates the accurate highlighting of infected regions achieved by the proposed SA-CB-BRSeg. Moreover, the incorporation of the pixel-wise distribution from SA-CB-BRSeg significantly enhances the segmentation of various stages of infected areas. The evaluation measures, ablation study, and the segmented maps visualization assessment provide strong evidence supporting the outperformance of the SA-CB-RESeg model. The proposed detection and segmentation CNNs have undergone comprehensive training and are now ready to be tested on previously unseen images. Additionally, the existing segmentation CNNs, both those trained from scratch and TL-based, are also subjected to analysis for comparative purposes.

The dataset utilized in this study primarily comprises 3D images, which are then transformed into a 2D image format. This transformation involves dividing each 3D CT lung into multiple 2D slices for analysis. Although the proposed framework is currently designed to handle 2D data, it is important to note that future development and optimization efforts will be directed towards specifically addressing applications involving 3D CT lung analysis. Furthermore, in medical challenges, the availability of labeled datasets is often limited. Therefore, to enhance the reliability of real-time diagnostics, we intend to apply the proposed framework to large-scale datasets^56^.

## Conclusions

COVID-19, an extremely transmissible illness, has had a profound global impact. These CT Lung images exhibit distinctive patterns associated with COVID-19 abnormalities. In this work, a new deep CNN-based two-stage diagnosis is developed to screen and diagnose COVID-19 infectious regions. This comprehensive methodology effectively learns the COVID-19 patterns by utilizing a range of characteristics such as consistent areas, textural changes, and borders. The proposed STM-BRNet is advantageous from data augmentation, TL-based diverse maps generations, and STM blocks. In addition, the method of residual learning employs a systematic feature extraction process by capturing fundamental image-level features and progressively incorporates more intricate texture-based distinctions. This innovative approach facilitates the acquisition of enhanced features at various levels of granularity. In contrast to existing deep CNNs, the STM-BRNet screening technique displays a significant discrimination capacity (98.11% F-score, 98.01% accuracy, and 98.12% recall). Our simulations have indicated that the SA-CB-BRSeg technique (with an IoU of 98.85% and DS of 96.40%) can accurately detect and analyze the infected areas in CT scans. The proposed SA-CB-RESeg benefited from training from scratch and fine-tuning COVID-19 data using TL and CB. The integrated methodology identifies the entire infected region of COVID-19, potentially assisting radiologists in evaluating the disease's stages of severity. The proposed framework has demonstrated a substantial performance improvement when compared to single-phase systems and other existing approaches. COVID-19 is a newly emerged disease that has limited labeled samples. Therefore, in the future, we will utilize the developed framework on big datasets to enhance the dependability of real-time diagnostics. Moreover, the dataset can be increased by augmenting the training data using GAN and labeling through SAM. Finally, it may be modified to segregate the infectious region into the multi-class challenge.

## Data Availability

The publicly available datasets used in this work that is accessible at https://zenodo.org/record/3757476#.Xpz8OcgzZPY, https://gitee.com/junma11/COVID-19-CT-Seg-Benchmark#datasets, https://medicalsegmentation.com/covid19/.
